# Prolonged shedding of severe acute respiratory syndrome coronavirus 2 in patients with COVID-19

**DOI:** 10.1080/22221751.2020.1852058

**Published:** 2020-12-10

**Authors:** Qian Li, Xiao-Shuang Zheng, Xu-Rui Shen, Hao-Rui Si, Xi Wang, Qi Wang, Bei Li, Wei Zhang, Yan Zhu, Ren-Di Jiang, Kai Zhao, Hui Wang, Zheng-Li Shi, Hui-Lan Zhang, Rong-Hui Du, Peng Zhou

**Affiliations:** a CAS Key Laboratory of Special Pathogens & State Key Laboratory of Virology, Wuhan Institute of Virology, Center for Biosafety Mega-Science, Chinese Academy of Sciences, Wuhan, People’s Republic of China; b University of Chinese Academy of Sciences, Beijing, People’s Republic of China; c Wuhan Municipal Health Commission, Wuchang Branch, Wuhan, People’s Republic of China; d Center for Biomedical Research, NHC Key Laboratory of Respiratory Disease, Tongji Hospital, Tongji Medical College, Huazhong University of Science and Technology, Wuhan, People’s Republic of China; e Wuhan Pulmonary Hospital, Wuhan, People’s Republic of China; f Joint Laboratory of Infectious Diseases and Health, Wuhan Institute of Virology & Wuhan Jinyintan Hospital, Wuhan, People’s Republic of China

**Keywords:** SARS-CoV-2, COVID-19, long-term carrier, prolonged shedding, transmission risk

## Abstract

Following acute infection, individuals COVID-19 may still shed SARS-CoV-2 RNA. However, limited information is available regarding the active shedding period or whether infectious virus is also shed. Here, we monitored the clinical characteristics and virological features of 38 patients with COVID-19 (long-term carriers) who recovered from the acute disease, but still shed viral RNA for over 3 months. The median carrying history of the long-term carriers was 92 days after the first admission, and the longest carrying history was 118 days. Negative-positive viral RNA-shedding fluctuations were observed. Long-term carriers were mostly elderly people with a history of mild infection. Infectious SARS-CoV-2 was isolated from the sputum, where high level viral RNA was found. All nine full-length genomes of samples obtained in March–April 2020 matched early viral clades circulating in January–February 2020, suggesting that these patients persistently carried SARS-CoV-2 and were not re-infected. IgM and IgG antibodies and neutralizing-antibody proﬁles were similar between long-term carriers and recovered patients with similar disease courses. In summary, although patients with COVID-19 generated neutralizing antibodies, they may still shed infectious SARS-CoV-2 for over 3 months. These data imply that patients should be monitored after discharge to control future outbreaks.

## Introduction

More than nine months since the first outbreak, severe acute respiratory syndrome coronavirus-2 (SARS-CoV-2) quickly spread to almost all countries in the world, resulting in over 50 million documented cases of coronavirus disease 2019 (COVID-19) infection and 1.2 million deaths (as of November 10, 2020) [1]. SARS-CoV-2 is mainly transmitted through the respiratory tract and causes pneumonia [2]. Following infection, the most common symptoms are fever (>37.3°C), fatigue, and a dry cough [2, 3]. To date, with limited success in terms of developing viral-specific drugs and vaccines, fast diagnosis and quarantine remain the most effective ways to stop SARS-CoV-2 from spreading.

Typically, patients with COVID-19 recover from infection within two weeks, particularly those with mild cases [2, 3]. However, several reports have shown the prolonged presence of viral nucleotides (nts) in the upper or lower respiratory tract, lung tissues, or intestinal tract (fecal matter) in recovered patients [4–7]. Likewise, an estimated range of 10–20% recurrent positivity for viral nt was also reported [7]. SARS-CoV-2 RNA was still detected in nasopharyngeal swabs approximately 51 days after the onset of symptoms in a clinically recovered patient [5]. It has been debated whether an extension of the quarantine period, currently 14 days in China, is necessary for recovered patients. Isolating infectious viruses from recurrent or persistent carriers has not been successful, raising the possibility that viral RNA positivity in such carriers only represents “dead viruses.” Similarly, most documented prolonged carriers or recurrently positive patients appear to be asymptomatic or have mild cases, and their transmission risk is largely unclear [4, 6]. However, a large amount of viral particles was found in a ready-for-discharge patient with COVID-19 in a postmortem pathologic study [8], suggesting that it is possible to carry live SARS-CoV-2 for a prolonged period. For example, a small-scale outbreak in Wuhan, China in May 2020 was sourced from a long-term carrier, and this could happen in other countries where the initial outbreak was controlled [9]. Currently, there is a lack of understanding in terms of the clinical, virological, and immunological features and transmission risks of long-term carriers. These knowledge gaps present difficulties in managing recovered patients.

## Methods

### Case definition and data collection

Patients with COVID-19 were normally discharged upon two consecutive negative reverse transcriptase-polymerase chain reaction (RT–PCR) tests, followed by a 14-day quarantine period in China. The presence of SARS-CoV-2 viral RNA was tested after two weeks of monitoring before the patients were released (negative results) or recharged (recurrent positive results). However, some patients positive for the virus lacked obvious disease symptoms. A group of 38 patients who recovered from acute infection, yet still shed viral RNA for over a month, was transferred to Wuhan Pulmonary Hospital for more specialized treatment. Those patients were referred to as long-term carriers in this study.

The patients’ clinical symptoms, viral loads in oral swabs (OSs) and sputum samples, routine blood parameters, and chest computed tomography (CT) results were monitored weekly at Wuhan Pulmonary Hospital, from March 11 to April 30, 2020. On April 20, samples were collected for in-depth virological testing, including virus isolation, genome sequencing, and neutralizing and non-neutralizing antibody titer testing at the Wuhan Institute of Virology. Viral RNA test results were compared to those from a group of patients in the acute stage in January 2020. Viral-antibody profiles were compared to a group of recovered patients (>28 days) with similar disease courses. We obtained medical records and compiled data for hospitalized patients, from the time of their first admission to April 30, 2020.

### Detection of SARS-CoV-2 viral RNA

RNA was extracted and tested by real-time RT–PCR with SARS-CoV-2-specific primers and probes (DAAN gene). The tests were carried out in biosafety-level 2 facilities at Wuhan Pulmonary Hospital or at the Wuhan Institute of Virology. A case was considered laboratory-confirmed if two targets (ORF1b or E) tested positive by real-time RT–PCR. A cycle threshold (Ct) value of <40 was defined as a positive test result.

### Virus isolation from sputum samples

Vero E6 cells cultured in Dulbecco's modified Eagle's medium containing 10% fetal bovine serum were used for virus isolation. All cells tested negative for mycoplasma contamination and were submitted for species identification and authentication by morphological microscopic evaluation. Sputum samples were diluted 1:1 with phosphate-buffered saline, vortexed, and then centrifuged at 13,000 × *g* for 10 min. Each supernatant was then incubated with Vero E6 cell monolayers. The cells were incubated at 37°C and observed daily for cytopathic effects. The culture supernatants were examined for the presence of SARS-CoV-2 by quantitative RT–PCR, and the cells were examined by immunofluorescence microscopy using an anti-SARS-CoV-2 NP antibody generated in-house (1:1,000) [10].

### Full-length genome sequencing and phylogenetic analysis

Viral RNA genome sequences were determined by next-generation sequencing (NGS). Sequencing libraries were constructed with the ATOPlex SARS-CoV-2 Full Length Genome Panel V1.0 (MGI, BGI-Shenzhen, China), following the manufacturer's instructions. NGS reads were assembled into genomes using Geneious (version 10.2.6) and MEGAHIT (version 1.2.9). Full genomes were aligned in ClustalW software (version 2.1) using a SARS-CoV-2 strain (WIV04, GenBank accession number MN996528.1) as the reference sequence [10]. Variation analysis was carried out mainly with BioEdit (version 7.1.3.0) and Clone Manager (Professional Suite, version 8). The results of the variation analysis were displayed using Circos (version 0.69–8) and Adobe Illustrator CC 2018. The viral clade was determined based on information deposited in the Global Initiative on Sharing All Influenza Data (GISAID) database (https://www.gisaid.org). A total of 1,327 complete SARS-CoV-2 genomes with high coverage and diverse collection dates from six different clades were randomly selected and downloaded from the GISAID database, and were aligned with nine patient genomes determined in this study using MAFFT (v7.407). An ML phylogenetic tree was constructed on CIPRES (http://www.phylo.org/index.php) using RAxML-HPC2 on XSEDE (8.2.12) and modified in FigTree (v1.4.3).

### Serology test

Serum IgM or IgG antibodies against the SARS-CoV-2 spike protein receptor-binding domain (RBD) were measured using an enzyme-linked immunosorbent assay (ELISA) kit generated in-house. Virus-neutralization assays were performed using the above reference SARS-CoV-2 strain in Vero E6 cells or the cPASS SARS-CoV-2 Neutralization Antibody Detection Kit (GenScript, catalog #L00847) [10].

### Statistical analysis

Continuous variables were expressed as medians and interquartile ranges or simple ranges, as appropriate. Data analyses were performed using SPSS, R software (version 3.6.2; Foundation for Statistical Computing), or GraphPad Prism software (version 6.0). Statistical analysis was performed using a two-tailed Student's t-test, and 95% confidence intervals were determined. *P* values less than 0.05 were considered to reflect statistically significant differences.

### Ethical approval

The ethics committees of the designated hospitals for emerging infectious diseases approved all procedures used to obtain human samples. Informed consent was obtained from each patient.

## Results

### Clinical characteristics of long-term carriers

**Figure 1.**
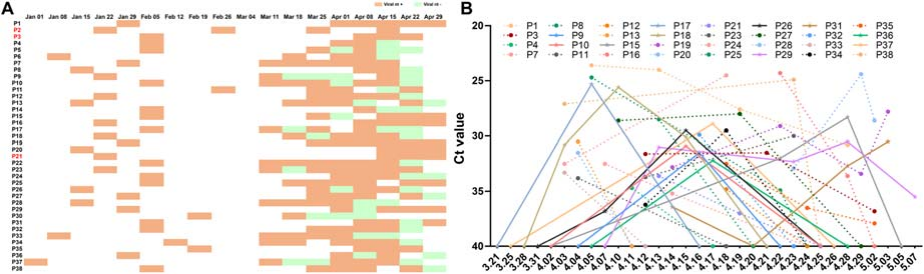
Carrying history and viral dynamics of long-term SARS-CoV-2 carriers. (A) Viral nt detection records of 38 patients with COVID-19 (P1–P38) from the day of admission to the end of April, 2020. Patients were transferred from multiple other hospitals to Wuhan Pulmonary Hospital in March 2020, where detection of the SARS-CoV-2 ORF1b and E genes in OSs was performed every week. Orange, viral nt-positive; green, viral nt-negative. (B) Viral RNA dynamics in OSs. Viral nt Ct values were missing for six patients and only the status (positive or negative) was recorded at certain time points for other patients. In the quantitative RT-PCR experiments, viral Cts of ≥40 were scored as negative results. The X-axis indicates the sampling date in 2020. Three severe cases are highlighted in red in Figure 1A (P2, P3, and P21).

Over 50,000 patients were enrolled in hospitals during the COVID-19 outbreak in Wuhan since late December 2019. Although the majority of these patients recovered, with SARS-CoV-2 nt-negative PCR results and an absence of clinical symptoms, a few patients remained positive for more than 30 days. At the end of the outbreak in Wuhan, 38 prolonged SARS-CoV-2 carriers were transferred to Wuhan Pulmonary Hospital from other hospitals during March–April 2020, where this investigation was conducted (Figure 1A). The median carrying history was 92 days from the time of the first admission, the longest carrying time was 118 days (P37, a 77 year-old male; Figure 1A), and the shortest carrying time was 58 days. Notably, some patients (*n* = 12, 31.6%) were identified as recurrent positive patients whose recurrent positive results probably resulted from viral load fluctuations in the respiratory tract (Figure 1B).

Most of the long-term carriers were older than 65 years (*n* = 20, 52.6%) (Table 1). Their clinical severities at the first admission were mainly mild (*n* = 35, 92.1%). During a 4-week observation period upon hospital transfer, 29 long-term carriers (76.3%) had no obvious clinical symptoms, such as fever, cough, or fatigue, which have been observed in most patients with COVID-19 [2]. Eight patients (21%) had mild symptoms, including a slight cough (*n* = 6, 15.8%; 5 also showed expectoration), a sore throat (*n* = 1, 2.6%), chest pain (*n* = 1, 2.6%), and occasional palpitations (*n* = 1, 2.6%), as shown in Table 1. However, chest CT results showed that all long-term carriers had signs of viral pneumonia-related lesions, including patchy shadows (*n* = 29, 76.3%), ground glass opacity (*n* = 21, 55.3%), or interstitial abnormalities (*n* = 3, 7.9%). Lymphocytopenia was only found in 4 patients (10.5%).

**Table 1.** Clinical characteristics of long-term SARS-CoV-2 carriers.

Characteristics	All patients (*N* = 38)
Age (years)	
Median (IQR)	65 (48.50–75.25)
30–49	10 (26.3%)
50–64	8 (21.1%)
>65	20 (52.6%)
Carrying history (days)	
Median (IQR)	92 (86.00–101.50)
Shortest	58
Longest	118
Disease severity upon admission	
Mild	35 (92.1%)
Serious	3 (7.9%)
Symptoms on day of study	
Fever	0 (0.0%)
Cough (expectoration)	6 (15.8%)
Fatigue	1 (2.6%)
Difficulty breathing	1 (2.6%)
Chest pain	1 (2.6%)
Sore throat	1 (2.6%)
Palpitations	1 (2.6%)
CT images examination	
Ground-glass opacity	21 (55.3%)
Patchy shadowing	29 (76.3%)
Interstitial abnormalities	3 (7.9%)
Lymphocyte count	
Median (IQR) −10*9/L	1.59 (1.22–1.92)
<1	*n* = 4 (10.5%)

### Infectious SARS-CoV-2 was isolated from long-term carriers

**Figure 2.**
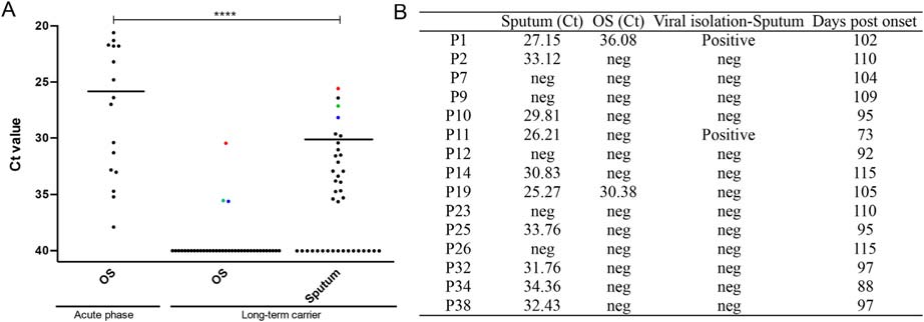
SARS-CoV-2 viral nt detection and virus isolation from long-term carriers. (A) Ct values for the E gene in RT-PCR assays using sputum or OS samples obtained from 38 prolonged SARS-CoV-2 carriers on April 20, 2020. The Ct values from long-term carriers were compared to those from OS samples collected from 17 patients with acute-phase COVID-19 on January 26, 2020. Median Ct values are shown only for the positive samples. The three positive OSs and the corresponding Ct values in the sputum are indicated. ***p < 0.001. (B) Results for virus samples isolated from selected sputum samples. The Ct values from the original samples are shown. Sampling date information (the number of days post-disease onset) is also shown. The patient numbering was described in Figure 1A.

Sputum and OS samples were collected for additional virological analysis (such as virus isolation) on April 20, 2020. The SARS-CoV-2-positive rate or viral RNA level was markedly higher in sputum samples (*n* = 21, 55.3%) than in OSs (*n* = 3, 7.9%), as shown in Figure 2A. However, the viral load of long-term carriers was still much lower than that of patients in the acute phase. In RT–PCR experiments, the median Ct was 32 for 21 sputum samples (scored as positive) from the long-term carries, which was significantly lower than the median Ct of 27 for 17 OSs obtained from patients in the acute phase.

Virus isolation was performed with sputum samples that showed higher viral positivity, and OSs were not tested due to a low level or absence of viral RNA. Two out of 16 (12.5%) sputum samples, which had Ct values of 27.15 and 26.21, were identified as SARS-CoV-2-positive using Vero E6 cells (Figure 2B). The samples were obtained from two patients who were first hospitalized on January 29, 2020 (P1) or February 26, 2020 (P11); thus, these samples were obtained 102 and 73 days after disease onset, respectively (Figure 1A). Neither patient had obvious disease symptoms except for lung abnormalities in CT scans on the day of sampling for virus isolation. Taken together, our data indicate that infectious SARS-CoV-2 viruses can still be isolated from some patients three months after the time of the first admission.

### Viral genome characteristics of long-term carriers

**Figure 3.**
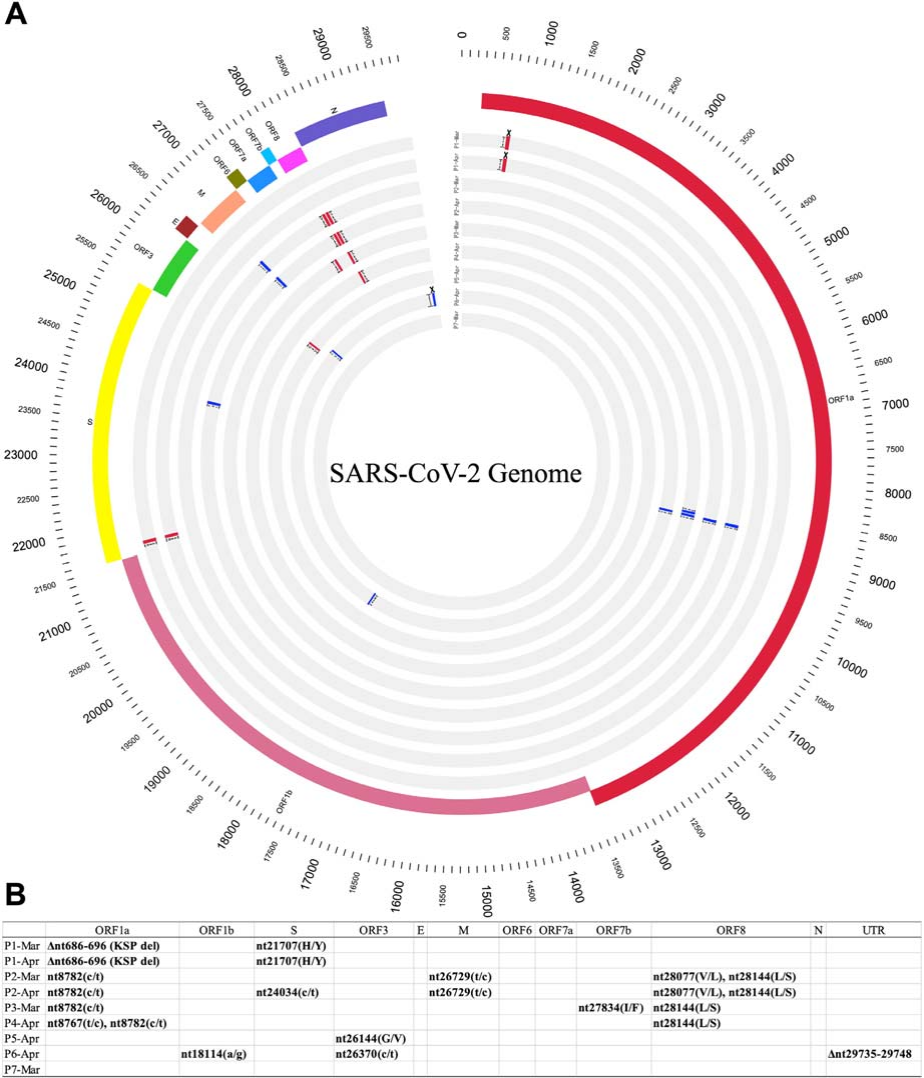
Viral genome characteristics of long-term SARS-CoV-2 carriers. Nine genomes obtained from sputum samples of seven patients (P1–P7) between March and April 2020 were compared to the reference genome sequence of SARS-CoV-2 strain WIV04 (GenBank accession number MN996528.1). Repeated sampling and sequencing were performed for P1 (March 29, 2020 and April 20, 2020) and P2 (March 31, 2020 and April 20, 2020). (A) Genome variations are shown using Circos plots, from the outer circle to the inner circle: SARS-CoV-2 genome length (bp), genome annotations, and nine SARS-CoV-2 genomes. Mutations refer to the reference WIV04 sequence: red text, non-synonymous change; blue text, synonymous change; crosses, deletion. All amino acid changes are indicated. A phylogenetic tree was also constructed and is shown in Supplementary Figure 1. (B) Summary of mutations detected. Capital letters indicate amino acid changes.

Arguments have been made that recurrent positive patients may be re-infected or that these long-term carriers may carry genetically unique viruses. To address these questions, we performed whole viral-genome sequencing for seven patients. By performing two consecutive sequencing analyses (separated by approximately 20 days) for two individuals, we found that both viral genome sequences were identical for patient 1 and that only one non-synonymous change occurred in patient [2]. All nine genomes shared high genetic identity with the WIV04 reference genome, which was obtained on January 2, 2020 (Figure 3). Phylogenetic analysis showed that all genomes clustered with early L, S, or V clades, suggesting that they were from January–February, 2020 (Supplementary Figure 1). Taken together, our data suggest that these patients more likely carried the same virus for a long term, rather than being re-infected. The long-term persistence of the virus was not limited to any genetically unique virus type or clade.

### Long-term carriers generated high titers of neutralizing antibodies

**Figure 4.**
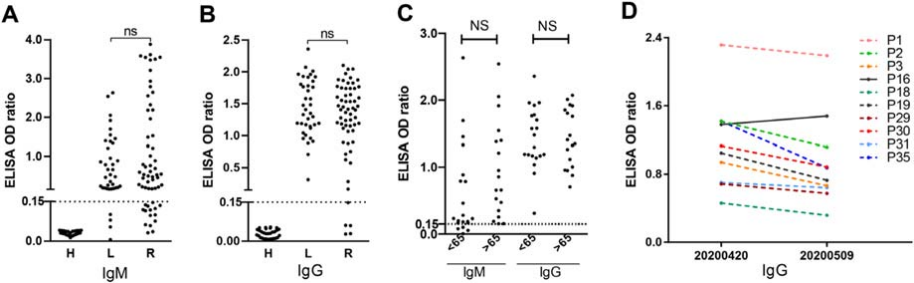
Antibody profiles. IgM (A) and IgG (B) antibody levels were compared between a group of long-term carriers (L, *n* = 38), a group of patients who recovered from COVID-19 (R, *n* = 60) who shared similar disease time courses, or a group of healthy donors (H, *n* = 30). A recombinant SARS-CoV-2 RBD protein was detected as the antigen in ELISA tests. OD_450_ values are shown. NS, non-significant. Neutralizing titers of serum samples from long-term carriers were tested and are shown in Supplementary Table 1. (C) Comparison of antibody levels in long-term carriers in different age groups. Patients were grouped, depending on whether they were younger or older than 65 years old. (D) Kinetics of antibody responses in long-term carriers. Ten patients were followed for antibody detection 20 days after the investigation on April 20. Their IgG antibody levels are shown.

Lastly, we tested antibody responses in long-term carriers. Convalescent sera from a group of 60 patients who had recovered for at least 28 days, as well as a group of healthy donors, were used for comparison purposes. The disease course of the recovered patients was comparable to that of the long-term carriers. The recombinant SARS-CoV-2 RBD protein, the main target of the neutralizing antibody, was used as the detection antigen. We observed that both groups of patients maintained high levels of IgM or IgG antibodies. No significant difference was found between the two groups (Figure 4A and 4B). In addition, no significant difference between either age group, i.e. younger or older than 65 years old (Figure 4C). Furthermore, 10 long-term carriers were followed for their antibody levels 20 days later. As observed in Figure 4D, 9 out of 10 patients showed decreased antibody responses.

Finally, we compared neutralizing titers using the commercial cPASS SARS-CoV-2 Neutralization Antibody Detection Kit. We tested all serum samples from long-term carriers (*n* = 38) and 15 samples from recovered patients. The results showed that an optical density at 450 nm (OD_450_) in RBD ELISA experiments of >1.20 was predictive of a neutralizing titer >1:80 in recovered patients, and a similar pattern was found with long-term carriers (Table S1 and S2). In addition, neutralizing titers did not differ significantly between the viral RNA-positive and RNA-negative long-term carriers (Table S1). Overall, the neutralizing-antibody profiles were also similar between long-term carriers and recovered patients.

## Discussion

To our knowledge, this is the first study of a group of prolonged SARS-CoV-2 carriers and, thus, has important public health significance in many ways. First, although long-term carriers generated neutralizing antibodies, they may still carry infectious viruses, although they may pose a lower risk for disease spreading than patients in the acute phase. Second, patients can carry the virus for as long as 118 days, which illustrates the importance of long-term monitoring of recovered patients.

It is generally believed that pathogenic coronaviruses normally cause acute infection, which results in either virus clearance or death in humans in a short time [11]. SARS-CoV-2 apparently can persist longer in some patients. Aged people with a history of mild infection, who may choose home isolation, could become long-term carriers and potentially serve as a source of future outbreaks. For example, a long-term carrier (>50 days carrying history) caused a small-scale outbreak in Wuhan in May 2020 [9]. It should be stressed that, although the viral load may fluctuate up and down, the risk posed by long-term carriers is still lower than that of patients during the acute phase. In the future, long-term monitoring of aged populations with previous mild infection histories by chest CT scans and viral nt detection appears to be necessary.

Our data also support the possibility that recurrent positive patients are actually long-term carriers. Viral load fluctuations were observed in many patients in this study, despite the fact that all patients were quarantined in the same hospital starting from March 2020, excluding the possibility of false testing by different technicians or re-infection from unknown sources. A plausible explanation for the negative detection is that the samples were collected during a down phase of viral replication, whereas detection during the rising phase may cause recurrent positivity. It has been argued that these observations of prolonged viral RNA positivity may only represent “dead viruses” in recurrently positive patients, since most previous reports only described the prevalence of RNA positivity [4, 5]. Our data clearly showed that infectious viruses could also be isolated from sputum. In addition, the viral genome clearly indicated that re-infection should not be the reason for long-term positive or recurrent positivity.

It is unclear why these patients could carry SARS-CoV-2 for such a long time, while having neutralizing antibodies. This phenomenon did not appear to be caused by a genetically distinct virus. A possible explanation is that the immune system maintained a balance with SARS-CoV-2. One location for targeting the virus is the lungs, as nearly all patients showed abnormalities in their chest CT scans. In support of this observation, a large amount of viral particles was still found in a ready-for discharge COVID-19 patient in a postmortem pathological study after the patient died from a sudden cardiovascular event [12]. Similarly, lung tissue from a patient with COVID-19 still showed weak SARS-CoV-2 RNA positivity, even though all other biological samples tested were found to be negative [8].

In summary, we show that long-term carriers may still show long-term shedding of infectious SARS-CoV-2, even if they have already generated neutralizing antibodies. We also provided virological, genomic, and antibody-based evidence that long-term carriers pose a transmission risk, although the risk was lower than that of patients in the acute stage of infection.

## Supplementary Material

Clean_copy_of_supplementary_materials.docx
