# Supplementary material for: Prolonged shedding of severe acute respiratory syndrome coronavirus 2 in patients with COVID-19
Source: Emerg Microbes Infect. 2020 Dec 10;9(1):2571–7. doi: 10.1080/22221751.2020.1852058 (PMC7734137; doi:10.1080/22221751.2020.1852058)
Supplement: Clean_copy_of_supplementary_materials.docx [file TEMI_A_1852058_SM4982.docx]

**Supplementary Data**

**Supplementary** **Table 1**. Neutralizing titers of long-term carriers (Long-term-P1-P38, red) and 15 recovered patients (Recovered-P1-P15, blue). Shown as neutralizing activity at each serum dilution. A higher than 20% blocking effect was considered as positive (highlighted in yellow), as indicated by cPASS kit manufactures instruction. The RBD IgG levels (related to Figure 4B, ELISA cutoff value=0.143) and viral nt levels in sputum (related to Figure 2A) was shown for each patient. Bottom table, neutralizing titer comparison between viral nt positive group and viral nt negative group. Patient numbering refers to Figure 1A. **^*^**virus was isolated from this patient.

| Patient No. | RBD IgG (OD450) | | | The minimum dilution tested of a positive result（≥20%） | | | | viral nt (sputum, Ct) | |
| --- | --- | --- | --- | --- | --- | --- | --- | --- | --- |
| Long-term-P32 | 2.357 | | | 1:160 | | | | neg | |
| Long-term-P13 | 2.072 | | | 1:160 | | | | 31.574 | |
| Long-term-P33 | 2.011 | | | 1:160 | | | | 34.646 | |
| Long-term-P5 | 1.962 | | | 1:160 | | | | neg | |
| Long-term-P35 | 1.960 | | | 1:80 | | | | 26.41 | |
| Long-term-P25 | 1.928 | | | 1:160 | | | | neg | |
| Long-term-P26 | 1.922 | | | 1:160 | | | | 35.27 | |
| Long-term-P9 | 1.915 | | | 1:160 | | | | neg | |
| Long-term-P12 | 1.851 | | | 1:160 | | | | neg | |
| Long-term-P10 | 1.804 | | | 1:160 | | | | neg | |
| *****Long-term-P22 | 1.780 | | | 1:80 | | | | neg | |
| Long-term-P6 | 1.766 | | | 1:160 | | | | 25.568 | |
| *****Long-term-P1 | 1.759 | | | 1:160 | | | | 27.134 | |
| Long-term-P27 | 1.702 | | | 1:160 | | | | neg | |
| Long-term-P15 | 1.579 | | | 1:80 | | | | neg | |
| Long-term-P8 | 1.576 | | | 1:80 | | | | 30.418 | |
| Long-term-P30 | 1.481 | | | 1:80 | | | | 32.89 | |
| Long-term-P7 | 1.474 | | | 1:160 | | | | neg | |
| Long-term-P17 | 1.407 | | | 1:80 | | | | 29.802 | |
| Long-term-P38 | 1.347 | | | 1:40 | | | | 33.773 | |
| Long-term-P37 | 1.325 | | | 1:80 | | | | neg | |
| Long-term-P14 | 1.295 | | | 1:40 | | | | 35.371 | |
| Long-term-P2 | 1.263 | | | 1:80 | | | | 32.901 | |
| Long-term-P3 | 1.193 | | | 1:40 | | | | 31.029 | |
| Long-term-P19 | 1.191 | | | 1:40 | | | | neg | |
| Long-term-P28 | 1.187 | | | 1:40 | | | | 35.621 | |
| Long-term-P29 | 1.170 | | | 1:80 | | | | neg | |
| Long-term-P24 | 1.169 | | | 1:40 | | | | 33.882 | |
| Long-term-P23 | 1.141 | | | 1:80 | | | | neg | |
| Long-term-P21 | 1.117 | | | 1:40 | | | | 34.711 | |
| Long-term-P34 | 1.039 | | | 1:20 | | | | neg | |
| Long-term-P4 | 1.006 | | | 1:80 | | | | neg | |
| Long-term-P16 | 0.957 | | | 1:80 | | | | 33.341 | |
| *****Long-term-P11 | 0.952 | | | 1:20 | | | | 28.159 | |
| Long-term-P31 | 0.910 | | | 1:20 | | | | neg | |
| Long-term-P20 | 0.879 | | | 1:80 | | | | 32.136 | |
| Long-term-P18 | 0.709 | | | 1:20 | | | | 29.621 | |
| Long-term-P36 | 0.312 | | | <1:20 | | | | 31.505 | |
| Recovered-P1 | 2.101 | | | 1:160 | | | | neg | |
| Recovered-P2 | 2.040 | | | 1:160 | | | | neg | |
| Recovered-P3 | 1.906 | | | 1:160 | | | | neg | |
| Recovered-P4 | 1.825 | | | 1:160 | | | | neg | |
| Recovered-P5 | 1.772 | | | 1:80 | | | | neg | |
| Recovered-P6 | 1.737 | | | 1:160 | | | | neg | |
| Recovered-P7 | 1.551 | | | 1:160 | | | | neg | |
| Recovered-P8 | 1.465 | | | 1:80 | | | | neg | |
| Recovered-P9 | 1.363 | | | 1:80 | | | | neg | |
| Recovered-P10 | 1.279 | | | 1:160 | | | | neg | |
| Recovered-P11 | 1.201 | | | 1:40 | | | | neg | |
| Recovered-P12 | 1.158 | | | 1:80 | | | | neg | |
| Recovered-P13 | 0.891 | | | 1:20 | | | | neg | |
| Recovered-P14 | 0.753 | | | 1:20 | | | | neg | |
| Recovered-P15 | 0.623 | | | 1:160 | | | | neg | |
|  | |  |  | |  |  |  | |  |
|  | | ≤1:20 | ≤1:40 | | ≤1:80 | ≤1:160 | Median | |  |
| Long-term all (n=38) | | 5 | 7 | | 13 | 13 | ≤1:80 | |  |
| viral nt+ (n=21) | | 3 | 6 | | 7 | 5 | ≤1:80 | |  |
| viral nt- (n=17) | | 2 | 1 | | 6 | 8 | ≤1:80 | |  |

**Supplementary** **Table 2**. Comparison of neutralizing titers indicated by cPASS kit and Plaque Reduction Neutralization Test (PRNT) in Vero E6 cells using SARS-CoV-2 virus. The minimum dilutions tested of a positive result were shown in the table below.

| Sample No. | cPASS (≥20%) | PRNT (≥50%) |
| --- | --- | --- |
| S1 | 1：90 | 1：2430 |
| S2 | 1：810 | 1：810 |
| S3 | 1：810 | 1：810 |
| S4 | 1：270 | 1：2430 |
| S5 | 1：30 | 1：90 |
| S6 | 1：90 | 1：810 |
| S7 | 1：270 | 1：2430 |
| S8 | 1：1350 | 1：1350 |
| S9 | 1：1350 | 1：1350 |
| S10 | 1：50 | 1：450 |
| S11 | 1：1350 | 1：12150 |

**Supplementary Figure 1**. **Phylogenetic analysis of SARS-CoV-2 genomes**. All genomes were downloaded from GISAID (https://www.gisaid.org), except the nine genomes obtained in this study (highlighted in red). Date of sampling was labeled for each genome. Sputum samples were collected and sequenced from seven patients on April 20. As comparison, two samples collected on earlier date, March 27 for P1 patient or March 31 for P2 patient, were also sequenced.
